# Regionalization, constraints, and the ancestral ossification patterns in the vertebral column of amniotes

**DOI:** 10.1038/s41598-022-24983-z

**Published:** 2022-12-23

**Authors:** Antoine Verrière, Nadia B. Fröbisch, Jörg Fröbisch

**Affiliations:** 1 Institut für Biologie, Humboldt-Universität zu Berlin, 10099 Berlin, Germany; 2 Museum für Naturkunde, Leibniz-Institut für Evolutions- und Biodiversitätsforschung, Invalidenstraße 43, 10115 Berlin, Germany

**Keywords:** Evolutionary developmental biology, Palaeontology

## Abstract

The development of the vertebral column has been studied extensively in modern amniotes, yet many aspects of its evolutionary history remain enigmatic. Here we expand the existing data on four major vertebral developmental patterns in amniotes based on exceptionally well-preserved specimens of the early Permian mesosaurid reptile *Mesosaurus tenuidens*: (i) centrum ossification, (ii) neural arch ossification, (iii) neural arch fusion, and (iv) neurocentral fusion. We retrace the evolutionary history of each pattern and reconstruct the ancestral condition in amniotes. Despite 300 million years of evolutionary history, vertebral development patterns show a surprisingly stability in amniotes since their common ancestor. We propose that this stability may be linked to conservatism in the constraints posed by underlying developmental processes across amniotes. We also point out that birds, mammals, and squamates each show specific trends deviating from the ancestral condition in amniotes, and that they remain rather unchanged within these lineages. The stability of their unique patterns demonstrates a certain homogeneity of vertebral developmental constraints within these lineages, which we suggest might be linked to their specific modes of regionalization. Our research provides a framework for the evolution of axial development in amniotes and a foundation for future studies.

## Introduction

The vertebral column is the defining feature of all vertebrates and has long been the subject of special attention by developmental and evolutionary biologists, yet certain key aspects of its development and evolution remain unexplored. Thanks to a large body of research^1–3^, the way vertebrae are formed throughout ontogeny is generally well understood. Vertebrae ossify via endochondral ossification, meaning that embryonic cartilaginous frameworks progressively mineralize into fully ossified elements. Ossification starts at one or several centers of ossification inside the cartilage matrix and spreads from there until the matrix is entirely replaced by bone.

In amniotes, elements of the vertebral column undergo two stages of endochondral ossification. The first stage consists of the mineralization of the neural arch and pleurocentrum. As the intercentrum is lost early in amniote evolution^4^, we only consider the pleurocentrum here and refer to it as centrum for the purpose of this study. The paired elements of the neural arch arise from two ossification centers, one on each side, that eventually fuse dorsomedially. The mineralization of the centrum starts in a pair of ventrally located ossification centers^5^ and then spreads through the centrum. Eventually, centrum and neural arch come into contact forming the neurocentral suture. In most amniotes, this suture later closes and both elements merge into a single vertebral unit. The second stage of vertebral ossification is marked by the mineralization of the transverse processes and the spinous process.

**Figure 1:**
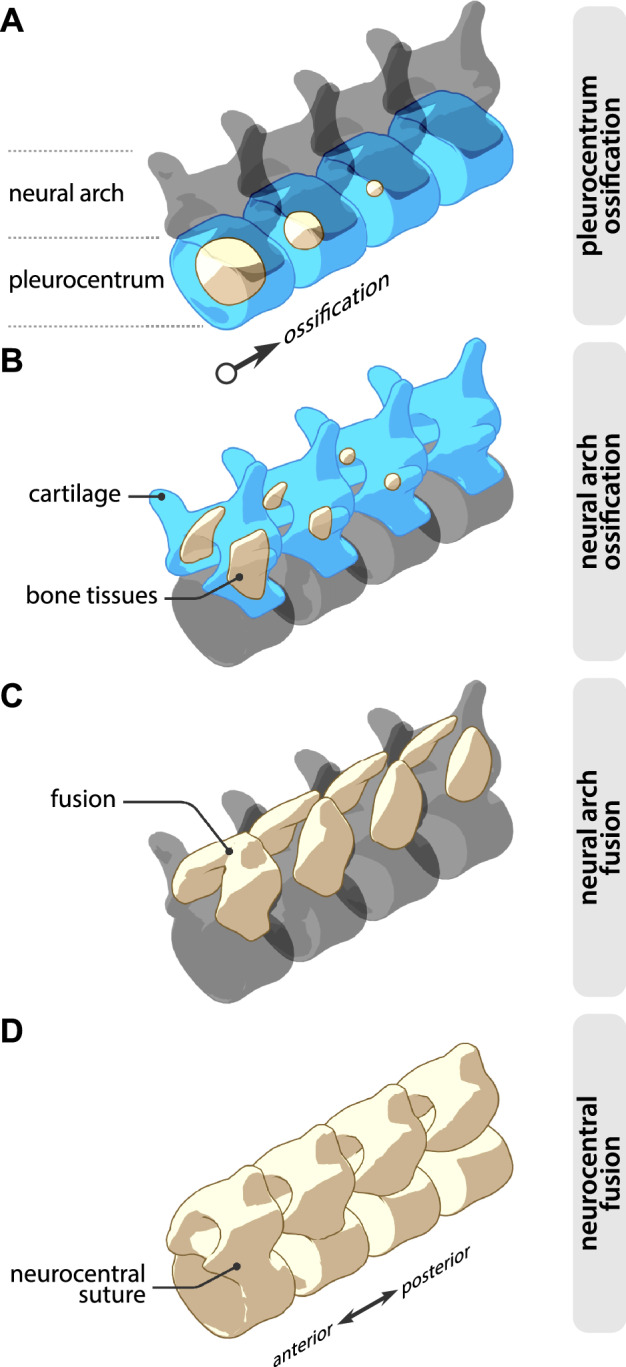
Schematic illustration of the four vertebral ossification patterns examined in the present study. (**A**) Pleurocentrum ossification. (**B**) Neural arch ossification. (**C**) neural arch fusion. (**D**) neuro central fusion.

Ossification events do not occur simultaneously throughout the vertebral column. On the contrary, they can originate in different locations along the vertebral column, occur at different times, and progress at different speeds and in different directions (see for instance^6–9^). In a given ossification pattern, it is possible to identify one or multiple points from which ossification spreads, whether it is the first vertebrae where ossification centers are visible or the first vertebrae in which given elements begin to fuse. For clarity, we will refer to these points as loci (Fig. 1). Loci are not to be confused with ossification centers, which are the spots where ossification begins within a single vertebra.

Although the sequence of ossification events leading to the formation of a single vertebra in amniotes has been extensively studied^8,10,11^, the timing of occurrence and spatial progression of these events along the vertebral column has rarely been documented. Even for model organisms, most studies only mention which of the neural arches or centra ossify first, if at all. With few exceptions^6,12^, the disparity between patterns of vertebral development in amniotes remains virtually unstudied, especially with respect to their evolutionary history. While this is partly due to the rarity of ontogenetic series in the fossil record, the axial column has also suffered from a limited research interest in developmental paleontology. Neurocentral fusion constitutes the only notable exception to this as it has been used as a proxy for maturity^13^, mostly in fossil archosaurs, especially in dinosaurs^14^. Consequently, this fusion pattern is relatively well documented for fossil members of this clade, albeit in a different context than in this study.

Vertebral elements are considered serially homologous within Amniota^15^, which allows for comparisons between clades. Here, we review the current state of knowledge on the four major patterns of primary ossification of the vertebral column (Fig. 1) in living and fossil amniotes: (i) the ossification of pleurocentra (ii) the ossification of neural arches, (iii) the fusion of paired neural arch elements, and (iv) neurocentral fusion. These patterns are easily observed on the skeleton and therefore more likely to be preserved in a fossil. In addition, thanks to some exceptionally well-preserved fossils of the early Permian parareptile *Mesosaurus tenuidens*, we provide the first documentation of vertebral ossification patterns in an early amniote. We compiled data on each of these patterns from the literature (Table S1) and used ancestral state reconstruction^16^ (ASR) to trace their evolutionary history in amniotes. Finally, we reconstructed the hypothetical ancestral condition for each of the patterns in amniotes.

Remarkably, we find that despite the 300-million-year history and great morphological and ecological diversity, axial ossification patterns seem to be relatively conservative within Amniota. Our results also show that each main amniote lineage evolved a specific pattern through acquisition and shifting of loci. We suppose these patterns may be linked to particular patterns of regionalization of the vertebral column in these large clades.

## Materials and methods

Our study focuses on four major patterns of axial ossification: centrum ossification, neural arch ossification, neural arch fusion, and neurocentral fusion. For each of these patterns, we reviewed the existing literature^6,8,14,17–63^ on extant and fossil amniotes and gathered information on two key aspects: the number of loci in the spine from where the patterns start and the position of these loci in the vertebral column (Table S1). Very little information was available on these patterns in the fossil record, as the axial skeleton of juvenile extinct amniotes has rarely been described in sufficient detail for the scope of our study.

Fortunately, based on exceptional specimens of the early Permian mesosaurid reptile *Mesosaurus tenuidens*, we were able to document patterns of axial ossification in one of the most basal clades of amniotes. Since these specimens are not embryos, we only have access to information on later stages of ossification. Therefore, our data on ossification patterns in *Mesosaurus* should not be considered a definitive description of ossification loci in the species, but rather an indication of whether patterns in the species match the ancestral condition reconstructed by our ASR.

In addition, to complete missing information on the patterns of some extant taxa, observations made on specimens from the collections of the Museum für Naturkunde in Berlin, Germany were included (Fig. S1). Variations in thickness and ossification in fossils were observed by thinly brushing the bones with acetone during investigation and photography, following the method described by Fröbisch et al.^64^. Advancement of fusion between vertebral elements was directly measured on fossils based on the degree of contact between elements and on the state of closure of sutures.

A composite tree of all studied species (Fig. S2) was constructed in Mesquite 3.7^65^ combining recently published phylogenies^66–80^. In addition to the taxa for which vertebral ossification data was available, fossil taxa were included in the tree to obtain a more accurate time-calibration following recommendations from the Fossil Calibration Database^81^. The resulting tree was time-calibrated based on occurrence dates from the Paleobiology Database^82^ and using equal time-scaling method. A subset of the time-calibrated tree was then generated for each of the four ossification patterns by trimming the original tree to match the taxon sample with available data.

In each studied taxon, ossification patterns were characterized in terms of number of loci and the position of these loci in the vertebral column. The presence/absence of a locus in these sections was scored as a binary character. In most cases, ossification and fusion do not begin in a single vertebra but rather in a series of adjacent vertebrae. If a series of fewer than 10 vertebrae showed the onset of ossification or fusion, the locus was identified as the midpoint of that range. For series with more than 10 vertebrae, no locus was scored for the pattern.

The presence or absence of a locus was noted in five sections of the vertebral column for each pattern: (i) cervical, (ii) upper dorsal/thoracic, (iii) lower dorsal/lumbar (iv) sacral, and (v) caudal. Each ossification pattern was translated into a matrix of five binary characters, one for each section of the vertebral column (Table S1). Binary characters were then tested for phylogenetic signal using Pagel’s λ^83^. Ancestral state reconstruction was performed on each character twice, once using maximum likelihood with symmetrical rates of evolution, and once using maximum parsimony with equal transition costs. To measure the effect of the new *Mesosaurus* data on the reconstruction, the analysis was also performed on a dataset excluding *Mesosaurus* with both maximum likelihood and parsimony.

Tree calibration and trimming, statistical analyses, and ancestral character reconstructions were performed in R v.4.0.5^84^ using the ape^85^, castor^86^, paleotree^87^, and phytools^88^ packages. Code in supplements (Dataset S1).

## Results

### Centrum ossification

**Figure 2:**
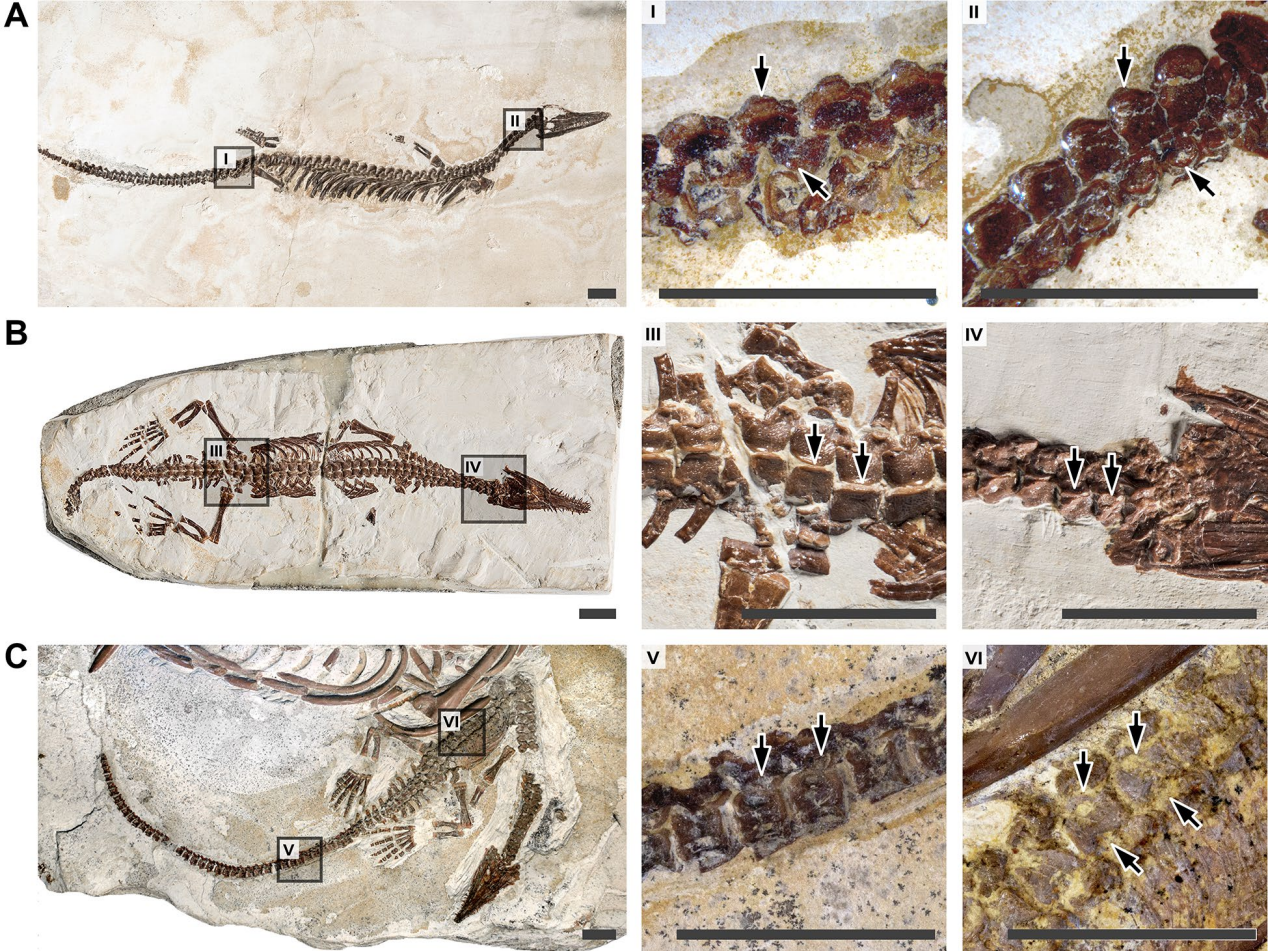
Juvenile specimens of *Mesosaurus tenuidens* showing axial ossification patterns. (**A**) SMF-R-4512, showing gradients of pleurocentrum and neural arch ossification; (**B**) BSPG 1979 I 37, showing a gradient of neural arch fusion; (**C**) MZSP-PV 1301, showing a gradient of neurocentral fusion. Specimens are oriented with the posterior region to the left and the anterior region to the right. Dark grey squares highlight the areas shown in close-up with the matching numbering. Arrows highlight differences in degree of ossification or fusion between two adjacent close-ups. Photographs (**A**) and (**B**) courtesy of (**C**). Radke (MfN). Photograph (**C**) courtesy of (**A**) Carvalho (MZSP). Scale bars: 10 mm.

**Figure 3:**
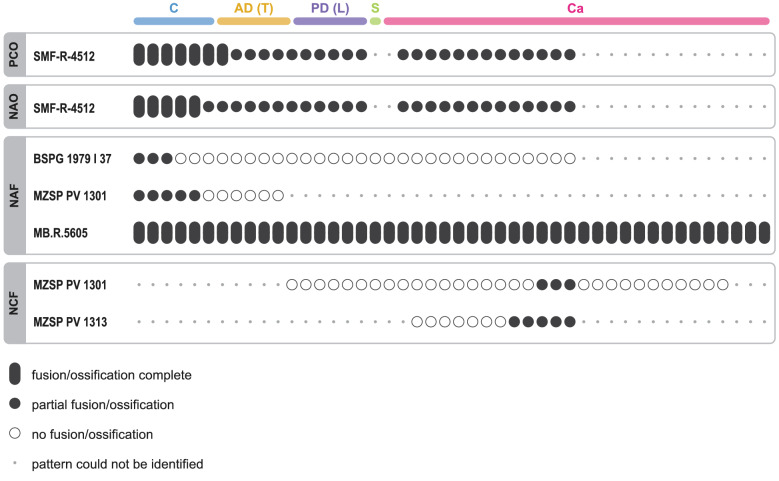
State of advancement of axial ossification patterns in juvenile *Mesosaurus tenuidens*. Each oval/circle represents two vertebrae. *PCO* pleurocentrum ossification, *NAO* neural arch ossification, *NAF* neural arch fusion, *NCF* neurocentral fusion. Vertebral sections are labelled as follow: *C* cervicals, *AD* anterior dorsals (scored as T in Table S1); *PD* posterior dorsals (scored as L in Table S1), *S* sacrals, C*a* caudals.

Indications of the sequences of vertebral ossification in the fossil reptile *Mesosaurus* were found in the juvenile specimen SMF-R-4512 (Fig. 2A). In this specimen, we could identify differences in the degree of ossification of some bones based on variations in hue and robustness. This correlation between appearance and ossification is well documented in a number of anamniote tetrapods with extensive ontogenetic series^64,89^. SMF-R-4512 shows a stronger coloration and thicker bone in the centra and neural arches of the cervical vertebrae as compared to other regions of the vertebral column (Fig. 2A), reflecting a more advanced ossification in this region. This suggests an anteroposterior gradient of centra and neural arch ossification in *Mesosaurus*, likely from a single anterior cervical locus (Fig. 3).

**Figure 4:**
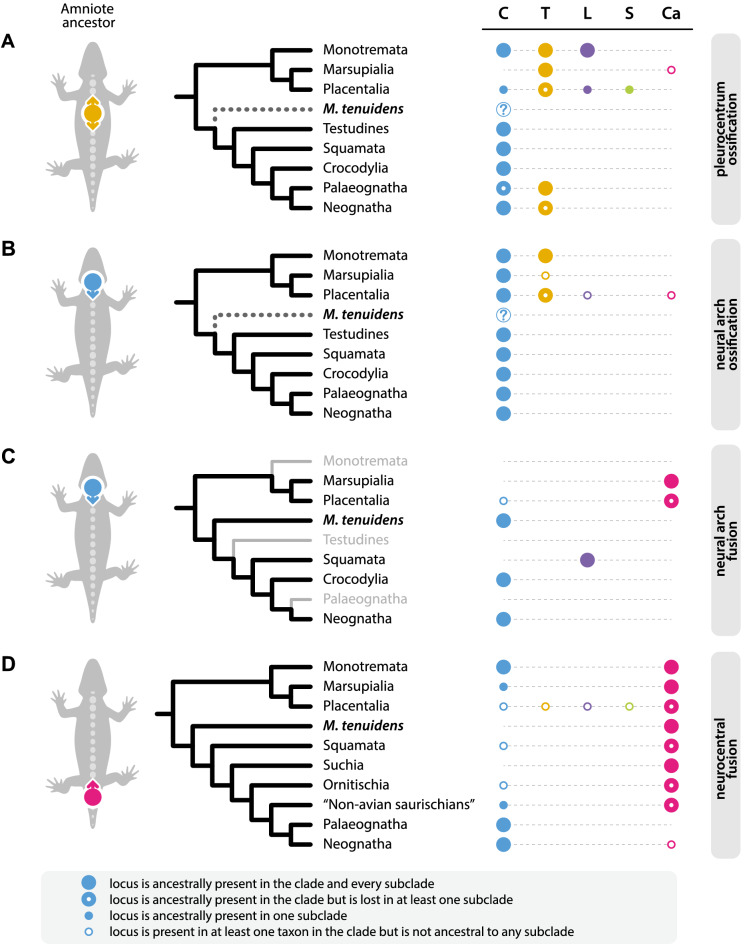
Distribution and ancestral state reconstruction of loci of axial ossification and fusion in amniotes. Colored circles on the grey silhouette represent the reconstructed ancestral condition in amniotes and arrows show the direction of ossification/fusion from each locus. *M. tenuidens* is excluded from the reconstruction for (**A**) and (**B**), but is included in (**C**) and (**D**). (**A**) Centrum ossification; (**B**) neural arch ossification; (**C**) neural arch fusion; (**D**) neurocentral fusion. Distribution of loci in tip taxa using the same color code. *C* cervical, *T* thoracic/anterior dorsal, *L* lumbar/posterior dorsal, *S* sacral, *Ca* caudal. Source data in Figs. S1–S4.

Centrum ossification is the only axial ossification pattern we reconstruct as potentially having two loci in amniotes ancestrally: one in the cervicals and one in the thoracics (Fig. 4A). While support for an ancestral thoracic locus is quite strong under both ASR models used, support for the ancestral cervical locus is much weaker with maximum likelihood (Fig. S3) than with parsimony (Fig. S4) and even drops completely under maximum likelihood when excluding *Mesosaurus* from the analysis (Fig. S5). Considering all models, our results hint towards the ancestral presence of the cervical loci, but fossil data is lacking to paint a clearer picture of the ancestral amniote condition.

The cervical locus of centrum ossification appears to be quite common among amniotes. Out of the 41 documented taxa, 26 present a cervical locus (Table S1). All birds and non-avian reptiles have a cervical locus, but most mammals do not. In models where the cervical locus is reconstructed as most likely ancestral to amniotes, it is subsequently lost in mammals, with this loss happening more or less early in the history of mammals depending on the ASR model used: in Mammalia with maximum likelihood (Fig. S3), in Theria with parsimony (Fig. S4).

Virtually all mammals studied have a thoracic locus of central ossification, with the exception of *Mogera* and *Talpa* (Table S1). *Oryctolagus* presents an additional lumbar locus of ossification, and *Dasypus* an additional caudal locus. Additionally, six of the studied bird species (*Coturnix*, *Dromaiusi*, *Gallus*, *Rhea*, *Sterna*, and *Struthio*) also possess an additional locus of ossification in the upper dorsal region (Table S1), and this locus is reconstructed as ancestrally present in paleognathous birds (Fig. 4A). Some mammalian taxa (*Dasypus*, *Bradypus*, *Oryctolagus*, *Sus*, and *Tachyglossus*) reacquire the cervical locus after it was ancestrally lost (Fig. S3). Except for these taxa, the cervical locus is a phylogenetically constrained trait (λ = 0.888, p < 0.001) and does not seem very plastic. Thus, rather than completely disappearing, the genetic signal for a cervical locus might have been merely muted in mammals and later reactivated in the aforementioned taxa. The potential causes of this reactivation remain to be investigated, since there is no clear common denominator to these taxa.

The evolutionary scenario for the thoracic locus follows a reverse path to that of the cervical locus: it is ancestrally present in amniotes, lost in reptiles but retained in mammals. The thoracic locus appears to be already absent in *Mesosaurus*, which would suggest it may too have been lost very early on in the history of Reptilia. Later, the thoracic locus is reacquired in birds, but it is lost again in *Tarniopygia*, *Meleagris*, and Anatidae. Precociality in birds such as turkeys and ducks is associated with specific development patterns^21,22^, which may be linked to the absence of the thoracic locus, but neither does this explain why this locus is also lost in the altricial *Tarniopygia*, nor why it is present in other precocial birds like paleognaths or galliformes. Better resolution on ossification pattern variability in birds would be necessary to understand these losses.

The moles *Talpa* and *Mogera* are the only mammals lacking a thoracic locus and possessing a lumbar and a sacral locus instead (Table S1). In this case, the locus is located in the first lumbar, a little more posteriorly than in other mammals, and could be the result of a slightly shifted timing of ossification of the same overall area. However, the additional sacral locus found in the two moles is clearly distinct and unique. Prochel^90^ identified that ossification timing in Talpidae diverges significantly from the standard mammalian condition due to their fossorial lifestyle, and it is very likely that a divergence in timing would also affect axial ossification loci.

### Neural arch ossification

Much like centrum ossification, neural arch ossification in the mesosaur *Mesosaurus* appears to be progressing posteriorly, probably from a single cervical locus (Figs. 2A, 3). This cervical locus of neural arch ossification is present in all amniote taxa (Fig. 4B). Although it is sometimes found in association with other loci, the cervical locus is in fact the only locus that is present in all reptiles, birds, marsupials, as well as in the temnospondyl *Micromelerpeton* (Table S1).

Additional neural arch loci are only found within Mammalia. All placentals possess a second locus of neural arch ossification in the thoracic region, as does the monotreme *Tachyglossus* (Table S1). Notably, marsupials do not have any additional neural arch loci, with the exception of *Sminthopsis* (Table S1). *Dasypus*, *Homo*, *Meriones,* and *Rattus* also display a third neural arch locus in the lumbar region. Again, the absence of a clear common denominator between theses taxa and a lack of resolution renders further interpretations difficult. *Talpa* is the only species possessing a caudal locus of neural arch ossification, which similarly to centrum ossification might be due to their peculiar development associated with fossoriality.

Both maximum likelihood and parsimony ASRs recover the single cervical locus as ancestrally present in all clades of amniotes and as the plesiomorphic condition in Amniota itself (Fig. 4B). The thoracic locus is ancestral to all mammals and to placentals but is lost in marsupials (Figs. S3, S4). It is absent in all other non-mammalian amniotes (Fig. 4B).

### Neural arch fusion

Neural arch fusion is the least well documented of the four patterns we considered. To the best of our knowledge, it has only been described in 17 extant amniotes, and never in any fossil tetrapod. Here, we document the first occurrence of this process in an early amniote. We made our observations on three specimens of *Mesosaurus tenuidens*: juveniles BSPG 1979 I 37 and MZSP-PV 1301, and adult MB.R.5605. In the juveniles, neural laminae are dorsally fused in the cervical region while they are unfused in posterior parts of the body; in the adult, all neural arches are fused (Fig. 3). This shows that neural arches fuse dorsally along an anteroposterior gradient, closing like a zipper. This “zipper-like” pattern is best observed in BSPG 1979 I 37 (Fig. 2B).

The cervical locus is found in *Mesosaurus*, in all studied archosaurs, and in placental mammals *Bradypus* and *Homo*. The lumbar/lower dorsal locus is found in all studied squamates whereas the caudal locus is found in the studied marsupials as well as in *Mus*, and *Oryctolagus*. The maximum likelihood ASR model reconstructs the cervical locus as the ancestral condition in Amniota (Fig. 4C). The locus is lost in Mammalia but is retained in Sauropsida and subsequently lost in Squamata (Fig. 4C). Parsimony is inconclusive about which of the cervical or the caudal locus is ancestral to Amniota, although results are similar to maximum likelihood for its subclades. The lower dorsal locus is strongly supported as ancestral to Squamata under maximum likelihood, but parsimony is inconclusive. The caudal locus is reconstructed as ancestral in Mammalia and its subclades under both models.

When excluding *Mesosaurus* from the analysis, ASR models are inconclusive and do not reconstruct any locus as ancestral to Amniota or Sauropsida, but still support an ancestral caudal locus in Mammalia (Figs. S5, S6).

In contrast to centrum and neural arch ossification, all studied taxa only ever bear one locus of neural arch fusion located in the cervical, lumbar/lower dorsal, or caudal section of the vertebral column (Fig. 4C). Interestingly, these loci appear mutually exclusive since we found no taxon where both conditions overlapped. Moreover, all locus location scores show a very strong significant phylogenetic signal (Fig. S3), meaning position and number of loci are not plastic traits. These results suggest that there is only a single neural arch fusion locus in the amniote vertebral column that would have shifted to different vertebral regions in some clades. This hypothesis implies the existence of transitional forms between locus positions, although such forms are yet to be discovered.

### Neurocentral fusion

Our observations on the neurocentral fusion of *Mesosaurus* are based on juvenile specimens MZSP-PV 1301, MZSP-PV 1313, on subadult PIMUZ A/III 591, and on adult specimens. The posterior half of MZSP-PV 1301 reveals that centra and neural arches are unfused in dorsal vertebrae, whereas the posteriormost caudals are partially fused (Figs. 2C, 3). In MZSP-PV 1313 a wider range of caudals is partially fused, and in adult specimens all neurocentral sutures are completed fused. This suggests the existence of a caudal locus of neurocentral fusion in mesosaurs.

In contrast to the three other ossification patterns, the cervical locus is neither the most common nor the reconstructed ancestral condition for neurocentral fusion in amniotes. Instead, our analysis shows that neurocentral fusion ancestrally started from a caudal locus in Amniota (Fig. 4D). The caudal locus is maintained in almost every amniote taxon but is absent in most birds, as well as in a few mammals, squamates, and dinosaurs (Table S1, Fig. S3).

Birds are the only main clade to deviate from the ancestral condition by adopting a cervical locus of neurocentral fusion (Fig. 4D). Interestingly, the transition from a caudal to a cervical locus precedes the rise of birds as it already occurs among non-avian dinosaurs: the ornithomimosaurian *Nqwebasaurus* and the tyrannosaurid *Dilong* also possess a cervical locus (Table S1), suggesting that this locus appeared in Coelurosauria (Figs. S3, S4). Moreover, both the cervical and the caudal locus are present in *Dilong*, showing that both loci coexisted in early coelurosaurians until the caudal locus later disappeared in more derived avian clades.

Several mammals and dinosaurs acquired a cervical locus in addition to the caudal one (Table S1), whereas the squamates *Cyrtodactylus* and *Zootoca* possess only the cervical locus and have lost the caudal locus. This suggests that the presence of a cervical locus is a rather plastic trait that emerged independently multiple times. Thoracic, lumbar, and sacral loci are exclusively found in mammals *Bradypus*, *Homo,* and *Mus* where they co-occur with other loci. With the exception of birds, non-caudal loci appear seemingly randomly, showing low or non-significant phylogenetic signal. This suggests that variations from the ancestral caudal locus condition arise for species-specific reasons and are not maintained over time, but more data on modern and fossil taxa would be necessary to investigate this hypothesis further.

## Discussion

### Pattern variations in the main amniote lineages

Despite their evolutionary history spanning over 300 million years and their great diversity and disparity, amniotes are surprisingly conservative with respect to axial ossification patterns. This is also reflected in the significant phylogenetic signal recovered in each of the patterns (Figs. S3, S4). It is remarkable that in the vast majority of amniotes some loci as well as the directionality of axial ossification have been maintained since the initial evolution of Amniota (Fig. 4). This is particularly interesting considering the disparity of vertebral morphologies and functions in the clade, and the diverse patterns of axial regionalization that have been recognized^8,91–94^.

Although axial ossification patterns have been relatively stable in amniotes, there are often variations from the ancestral patterns. In fact, the main lineages of amniotes (mammals, birds, squamates) all deviate from the ancestral condition in at least one of the axial ossification patterns. Yet, these variations are not random. The major lineages show remarkable conservatism within themselves, and they are each characterized by specific trends.

Squamates show only very little variation internally and from the ancestral amniote condition, but they distinguish themselves by being the only lineage to possess a lower dorsal locus of neural arch ossification instead of a caudal one (Fig. 4C). Birds notably diverge from the ancestral amniote patterns by being the only lineage with a cervical locus of neurocentral fusion (Fig. 4D), but they also reacquire the thoracic locus of centrum ossification ancestrally lost in Sauropsida (Figs. 4A, S3).

Mammals feature the most deviations from the reconstructed ancestral condition for Amniota. They are the only clade to lose the cervical locus of centrum ossification plesiomorphically (Fig. 4D), the only clade to adopt a thoracic locus of neural arch ossification (Fig. 4B), and the only clade to switch from a cervical to a caudal locus of neural arch fusion (Fig. 4C). Moreover, axial ossification patterns within Mammalia show a lot of variation from the ancestral mammalian condition, more than in any other amniote clade. Co-occurrence, loss, or reacquisition of loci are fairly common, and some loci positions, especially in the lumbar and sacral area, are exclusively found in mammals (Fig. 4).

The existence of trends in major lineages provides an interesting basis for understanding the evolution of developmental constraints in amniotes. While the mechanisms driving the trends are unclear, there is a conspicuous parallel to be drawn between axial ossification patterns in amniote lineages and regionalization. Regionalization is the segmentation of the vertebral column into more or less distinct functional sections. Squamates, mammals, and birds each possess very specific regionalization profiles, from virtually identical presacrals in squamates^95^ to highly differentiated vertebral segments in mammals and birds^15,95^.

The current state of knowledge prevents us from further testing links between types of regionalization and axial ossification. Yet, recent works have highlighted the influence of vertebral developmental tempo and growth rates variations on the initiation of axial regionalization^96^, showing how these processes might be interrelated. Moreover, there are a few cases where changes in regionalization parallel modifications of axial ossification patterns. For instance, the transition from a caudal to a cervical locus of neurocentral fusion in coelurosaurian dinosaurs strikingly mirrors the progressive loss of articulation in tail vertebrae in favor of a fused pygostyle in birds, a loss probably caused itself by variations in *Hox* genes^97^. Similarly, the adoption of a fixed number of seven cervicals in mammals through *Hox* gene mutations coincides with the acquisition of an additional thoracic locus of neural arch ossification^11,98^.

While these examples are consistent with our hypothesis, and although the study of vertebral regionalization has expanded rapidly in recent years^8,92,95,96,99,100^, some amniote clades remain under-represented in terms of quantitative data on the topic: modern turtles and squamates, but also most fossil amniotes. Investigating vertebral regionalization and development in these taxa would be crucial to refine our understanding of the evolutionary history of the vertebral column in amniotes, and our study lays the foundations for future works on the topic.

### Axial ossification patterns and Hox genes

It seems likely that the observed conservatism of axial ossification patterns is due to constraints imposed by the underlying processes during vertebral development. Among these processes, the mechanical stresses to which the embryos are subjected can have an effect on ossification^40,101–103^. The application of mechanical stresses, particularly by muscle fibers, can trigger ossification. Axial ossification patterns could thus be driven by local mechanical stress variations during development and would be potentially more variable than our results seem to show. Our study is based on limited ontogenetic series, and which does not allow for observations on intraspecific variation or to test for the effect of mechanical stress during development in fossil taxa. However, the high stability of the patterns between groups and over time makes the hypothesis of a very high intra-clade variation rather unlikely. If mechanical stresses have an effect, then either this effect is negligible on these time and phylogenetic scales, or the mechanical stress fields during development are also phylogenetically constrained. In the latter case, they would contribute directly to the evolution of patterns we see in amniotes.

Genetic frameworks are another process that may control the appearance of ossification and fusion loci. While genetic material does not preserve in deep geological time, comparisons with modern representatives can provide some insights into the genetic underpinnings of axial patterning in fossil taxa^96,98,104,105^. In this context, the role of *Hox* genes and their respective regulators in directing axial patterning in vertebrates specifically and animals more broadly, has long been known^99,105–108^.

Moreover, a number of studies have shown that overlapping expression domains of *Hox* genes along the anteroposterior axis of the embryo directly impact vertebral morphologies in the adult^6,11,53,105,109–111^ and that the ranges of *Hox* gene expression domains can also influence the relative timing of ossification in vertebral elements^6,8,53^. Therein, certain *Hox4-10* paralogues linked to vertebral regionalization have been suggested to have been retained in Amniota since the last common ancestor of the clade^104^. Therefore, axial ossification models including those of fossil taxa may indeed reflect to some degree how developmental constraints prevented or channelized evolutionary innovation. However, the more detailed roles that *Hox* and other genes play in directing ossification and fusion patterns in extinct and extant taxa alike remains to be investigated.

### Scenarios for the evolution of axial ossification patterns

We propose the following ancestral condition for axial ossification patterns in amniotes: (i) pleurocentrum ossification proceeding posteriorly from two loci in the cervical and the thoracic region, (ii) neural arch ossification proceeding posteriorly from a single cervical locus, (iii) neural arch fusion progressing posteriorly from a single cervical locus in a “zipper-like” pattern, and (iv) neurocentral fusion proceeding anteriorly from a caudal locus. From this ancestral condition, their exact evolutionary development remains obscure due to the current lack of fossil data.

On the reptilian branch of Amniota, the exceptional specimens of *Mesosaurus* provide unique information, demonstrating how fossils can provide crucial insights for filling gaps in the data based on extant animals alone. For neural arch and neurocentral fusion patterns, *Mesosaurus* displays the reconstructed ancestral reptilian condition, whereas the observed ossification gradients of centrum and neural ossification are compatible with the reconstructed ancestral condition in reptiles and amniotes. The observations in this ancient amniote taxon are therefore consistent with our reconstruction.

On the mammalian branch, however, the lack of information on vertebral ossification patterns in non-mammalian synapsids is problematic. Axial ossification patterns have never been documented in any pelycosaur-grade synapsid, in therapsids, in Mesozoic or even Cenozoic mammals. Additional data from fossil synapsids would greatly help to refine the resolution and robustness of the reconstructed evolutionary history of these traits in amniotes.

Based on the current data and depending on what might eventually be found in synapsids in the future, we predict three scenarios: (i) fossil synapsids show patterns of axial ossification resembling those of reptiles. In that case, the observed reptilian patterns would reflect the ancestral condition in all amniotes. The condition observed in modern mammals would then constitute a synapomorphy adopted relatively late in their evolutionary history. This would support our hypothesis that the specific axial ossification patterns found in crown-mammals may be connected to the evolution of a stronger vertebral regionalization in therapsids^95^; (ii) fossil synapsids show patterns of axial ossification resembling those of crown-mammals. This would imply that the two main branches of Amniota each developed their own specific modes of axial ossification very early in their evolutionary history, potentially at the time of their original dichotomy. This would in return suggest that axial ossification patterns might have played a role in this dichotomy; (iii) fossil synapsids show unique patterns of axial ossification. This scenario would combine the other two, with a dichotomy in axial ossification patterns at the base of Amniota and the innovation of another, different condition in crown-mammals. Regardless of which scenario might be supported once additional data on fossil synapsids becomes available, it will yield important implications for the evolutionary history of morphological diversity in amniotes.

## Conclusions

Reviewing the literature and with additional data from exceptionally well-preserved fossils, we reconstruct the ancestral axial ossification and fusion patterns in amniotes:

- Centra ossify from neck to tail, starting from two loci in the cervical and the thoracic region.
- Neural arches also ossify posteriorly but from a single cervical locus.
- Neural arches fuse together in a “zipper-like” pattern, starting in a cervical locus.
- Neurocentral fusion begins in the caudal region.

Despite the long evolutionary history of amniotes, all four axial ossification patterns show a strong phylogenetic signal and appear to have been quite stable over time. We propose that this conservatism may be linked to constraints posed by underlying developmental processes across amniotes. Our study also highlights specific trends in the axial ossification of birds, mammals and squamates. We suggest a correlation between these trends and the respective vertebral regionalization patterns in these clades. This study provides a framework for understanding the evolution of axial development constraints in fossil and modern amniotes, while laying groundwork for future research on the topic.

## Supplementary Information

Supplementary Information 1.

Supplementary Figure S1.

Supplementary Figure S2.

Supplementary Figure S3.

Supplementary Figure S4.

Supplementary Figure S5.

Supplementary Figure S6.

Supplementary Table S1.

Supplementary Information 2.

## Data Availability

All data generated or analysed during this study are included in this published article and its supplementary information files.
