# Supplementary material for: Regionalization, constraints, and the ancestral ossification patterns in the vertebral column of amniotes
Source: Sci Rep. 2022 Dec 23;12:22257. doi: 10.1038/s41598-022-24983-z (PMC9789111; doi:10.1038/s41598-022-24983-z)
Supplement: Supplementary file 1 — Supplementary Information 1. [file 41598_2022_24983_MOESM1_ESM.docx]

**Regionalization, constraints, and the ancestral ossification patterns in the vertebral column of amniotes**

Antoine Verrière, Nadia B. Fröbisch, Jörg Fröbisch

# Supplementary Files

**Dataset S1.** R code and files used for the ancestral state reconstruction and the statistical analysis.

**Fig. S1.** Advancement of ossification and fusion in the ZMB taxa. Each oval/circle represents one vertebra. Vertebral sections are labelled as follows: C: cervical; T: thoracic/upper dorsal; L: lumbar/lower dorsal; S: sacral; Ca: caudal. Abbreviations used: PCO: pleurocentrum ossification; NAO: neural arch ossification, NAF: neural arch fusion, NCF: neurocentral fusion.

**Fig. S2**. Time-calibrated supertree of amniotes.

**Fig. S3**. Ancestral state reconstruction of the presence of a locus for pleurocentrum ossification in each section of the vertebral column, including *Mesosaurus* and using maximum likelihood. In Fig. S3 to S6: dark blue and white circles at tips respectively mark the presence and the absence of a locus in the section; pie charts at nodes show the probability of each state being ancestral for the clade; vertebral sections and patterns labelled as in Fig. S1.

**Fig. S4.** Ancestral state reconstruction of the presence of a locus for pleurocentrum ossification in each section of the vertebral column, using parsimony.

**Fig. S5.** Ancestral state reconstruction of the presence of a locus for neural arch ossification in each section of the vertebral column, using maximum likelihood.

**Fig. S6.** Ancestral state reconstruction of the presence of a locus for neural arch ossification in each section of the vertebral column, using parsimony.

**Table S1.** Position of ossification and fusion loci in the studied taxa. Sections of the vertebral column are labelled as follows: C: cervical; T: thoracic/upper dorsal; L: lumbar/lower dorsal; S: sacral; Ca: caudal. Black and white circles respectively mark the presence and absence of a locus in the vertebral region. Hyphens marks the absence of data for this pattern. n: number of specimens constituting the sequence.
