# Supplementary material for: Regionalization, constraints, and the ancestral ossification patterns in the vertebral column of amniotes
Source: Sci Rep. 2022 Dec 23;12:22257. doi: 10.1038/s41598-022-24983-z (PMC9789111; doi:10.1038/s41598-022-24983-z)
Supplement: Supplementary file 2 — Supplementary Figure S1. [file 41598_2022_24983_MOESM2_ESM.pdf]

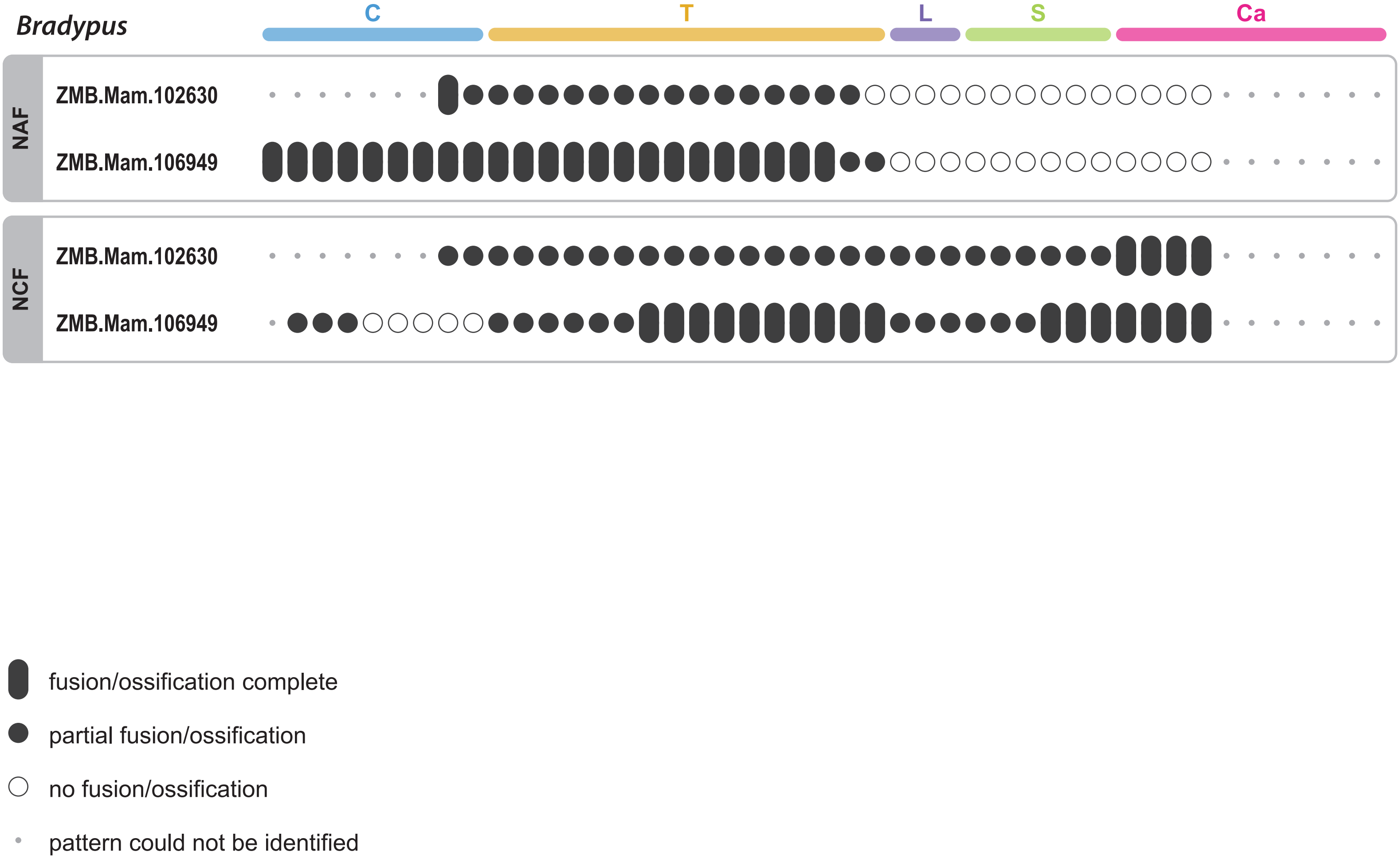

Bradypus
C
T
L
S
Ca
NAF
ZMB.Mam.102630
ZMB.Mam.106949
NCF
ZMB.Mam.102630
ZMB.Mam.106949
fusion/ossification complete
partial fusion/ossification
no fusion/ossification
pattern could not be identified

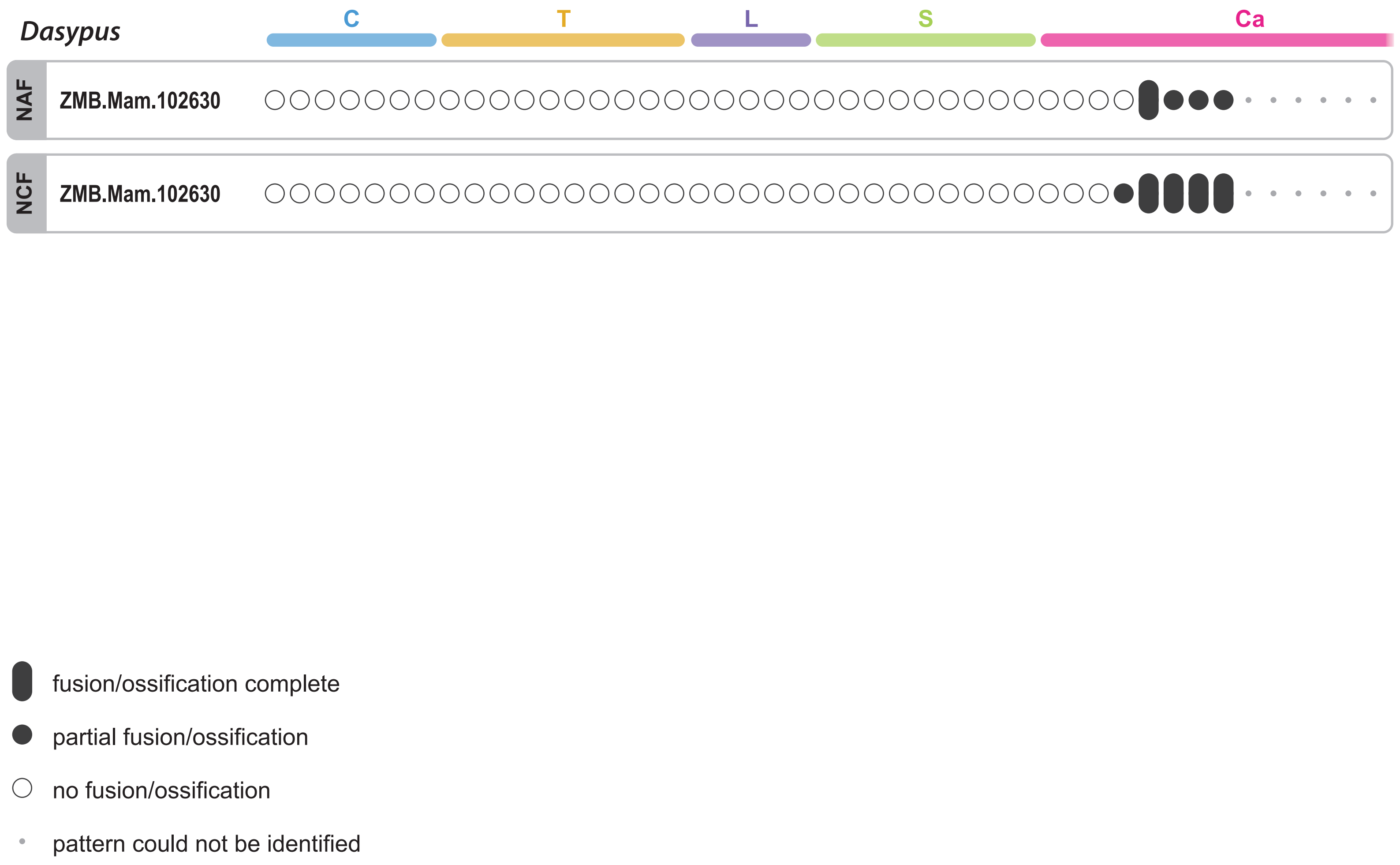

Dasypus
C
T
L
S
Ca
NAF
ZMB.Mam.102630
NCF
ZMB.Mam.102630
fusion/ossification complete
partial fusion/ossification
no fusion/ossification
pattern could not be identified

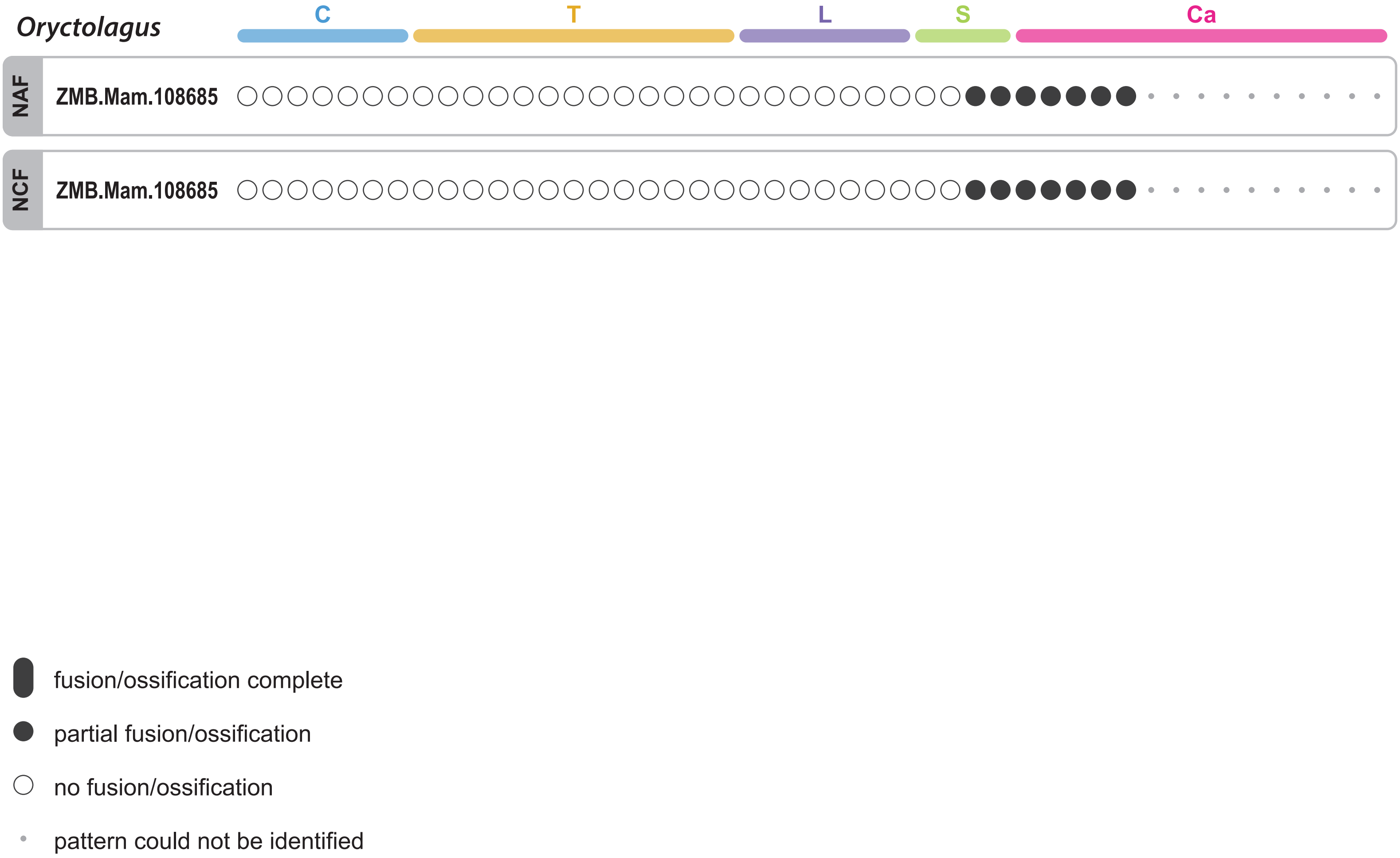

Oryctolagus
C
T
L
S
Ca
NAF
ZMB.Mam.108685
NCF
ZMB.Mam.108685
fusion/ossification complete
partial fusion/ossification
no fusion/ossification
pattern could not be identified

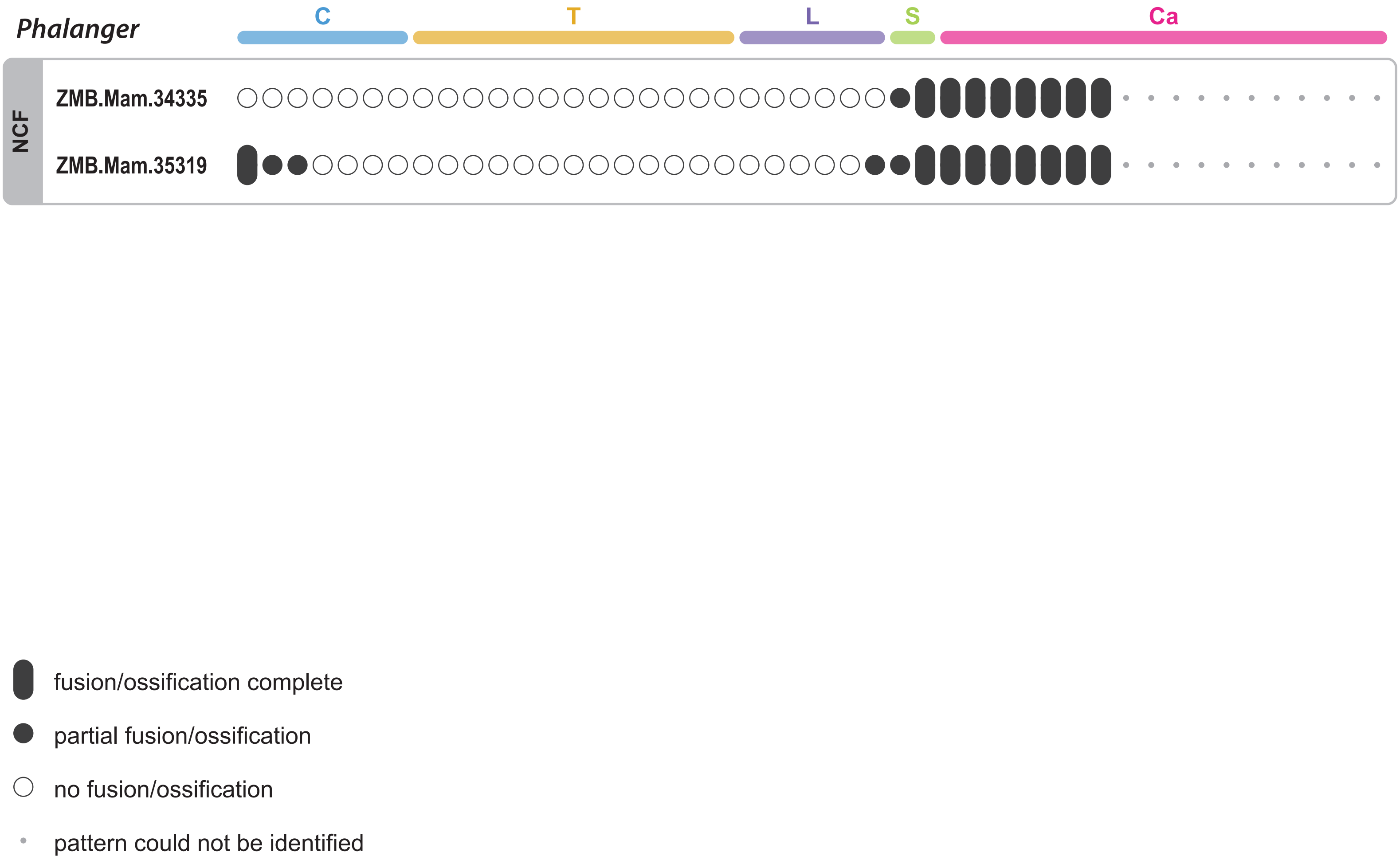

Phalanger
C
T
L
S
Ca
NCF
ZMB.Mam.34335
ZMB.Mam.35319
fusion/ossification complete
partial fusion/ossification
no fusion/ossification
pattern could not be identified

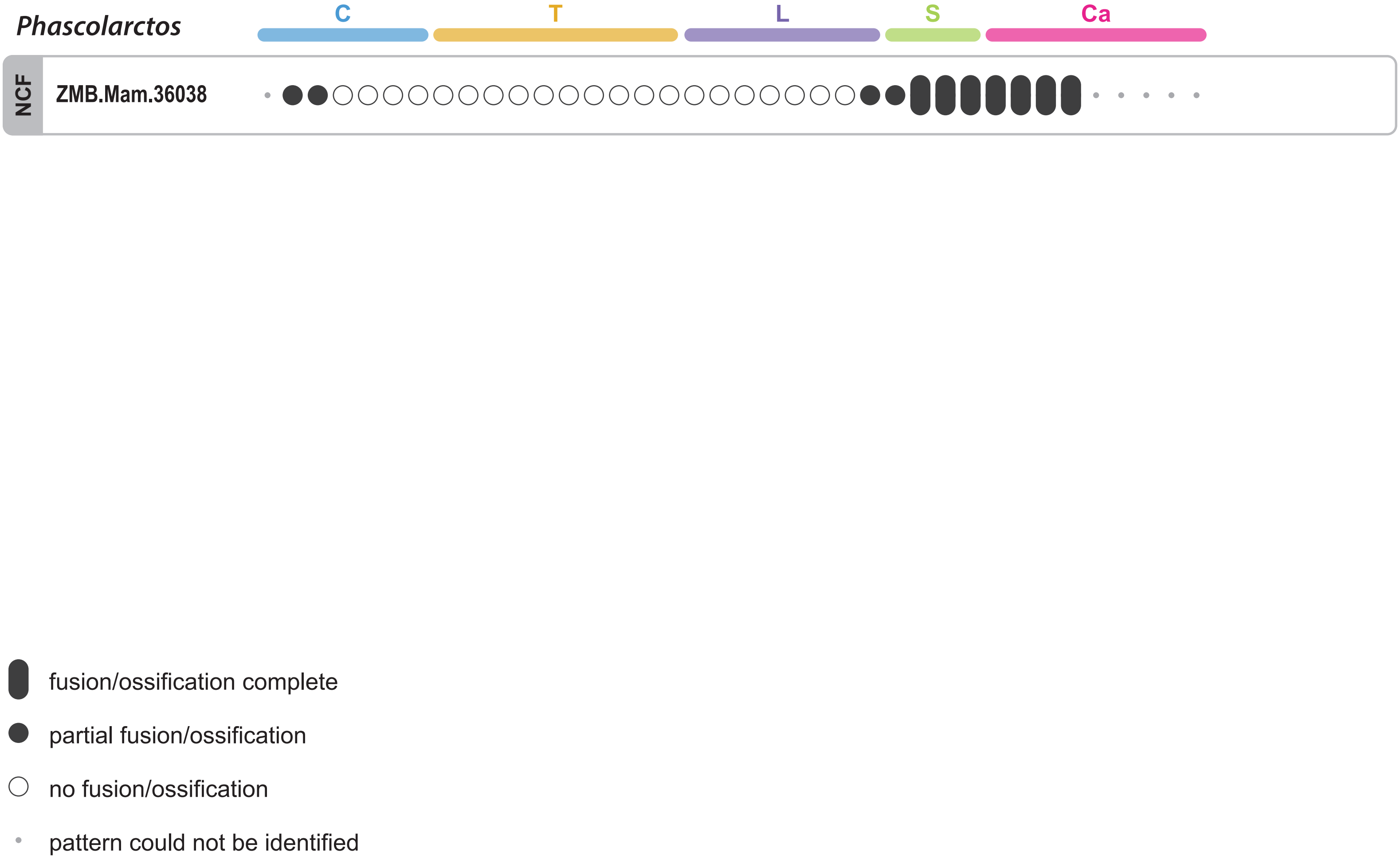

Phascolarctos
C
T
L
S
Ca
NCF
ZMB.Mam.36038
fusion/ossification complete
partial fusion/ossification
no fusion/ossification
pattern could not be identified

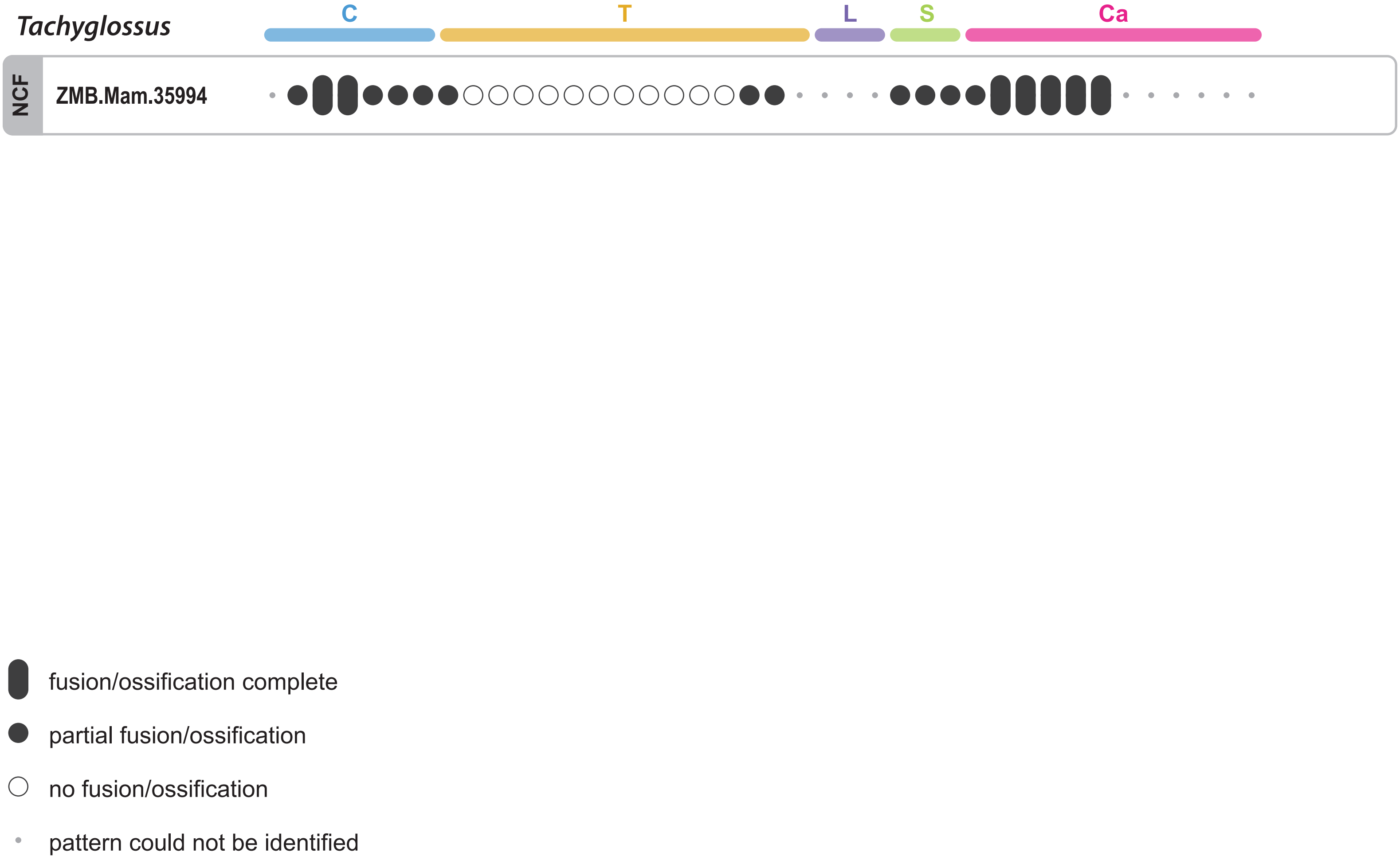
Tachyglossus
C
T
L
S
Ca
NCF
ZMB.Mam.35994
fusion/ossification complete
partial fusion/ossification
no fusion/ossification
pattern could not be identified
