# Supplementary material for: Regionalization, constraints, and the ancestral ossification patterns in the vertebral column of amniotes
Source: Sci Rep. 2022 Dec 23;12:22257. doi: 10.1038/s41598-022-24983-z (PMC9789111; doi:10.1038/s41598-022-24983-z)

PCO - Mesosaurus: removed - maximum likelihood

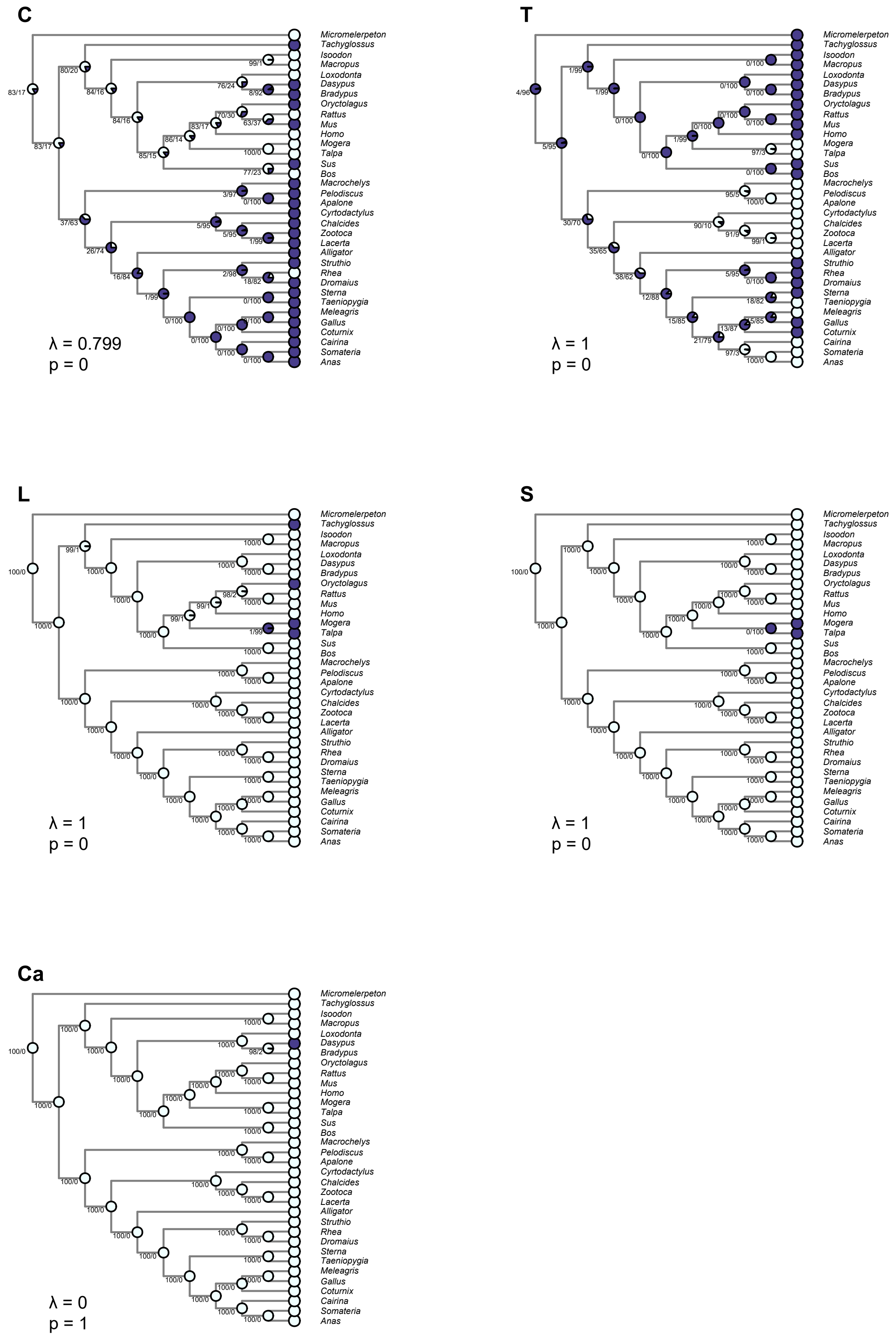

NAO - Mesosaurus: removed - maximum likelihood

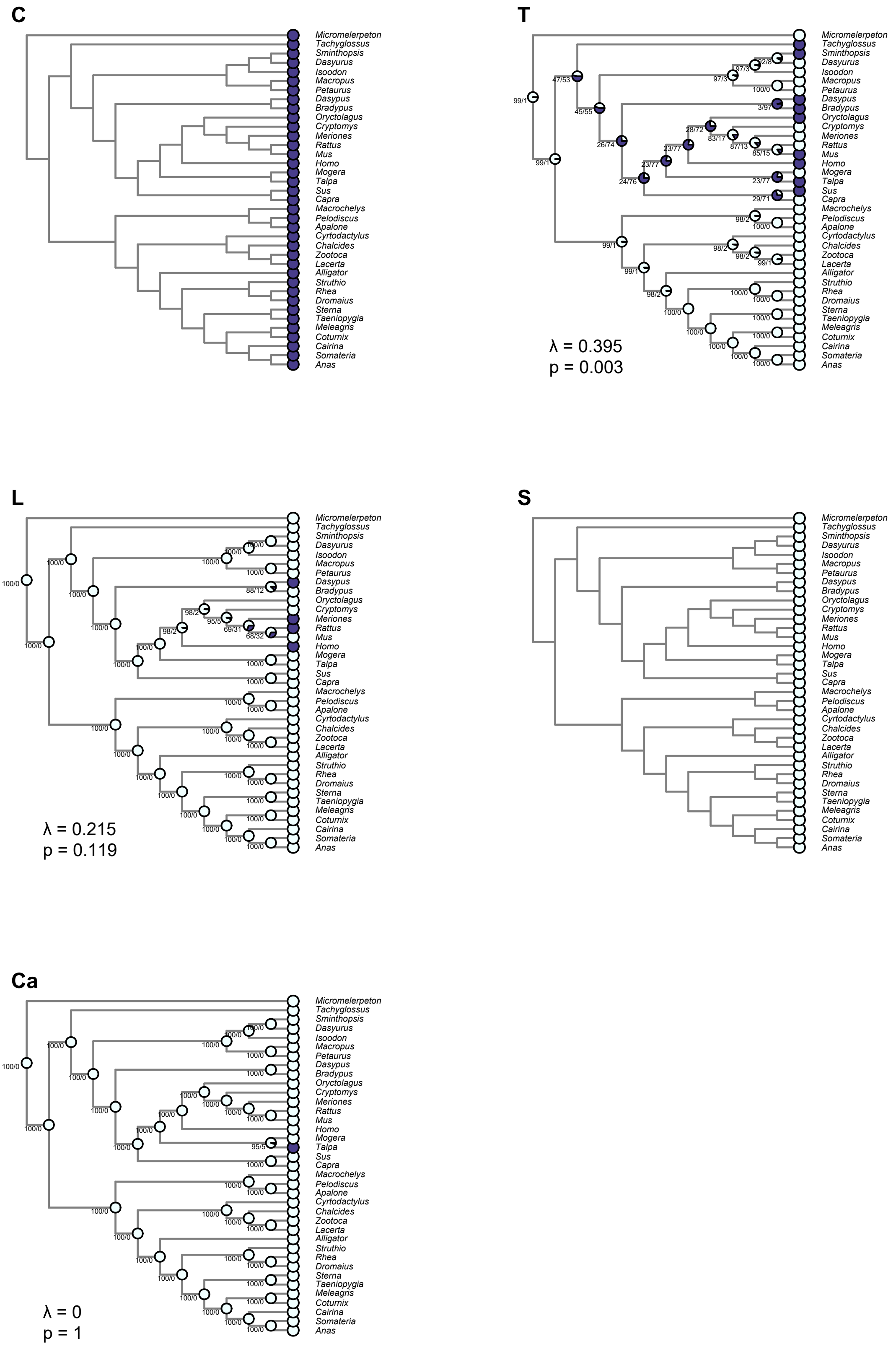

NAF - Mesosaurus: removed - maximum likelihood

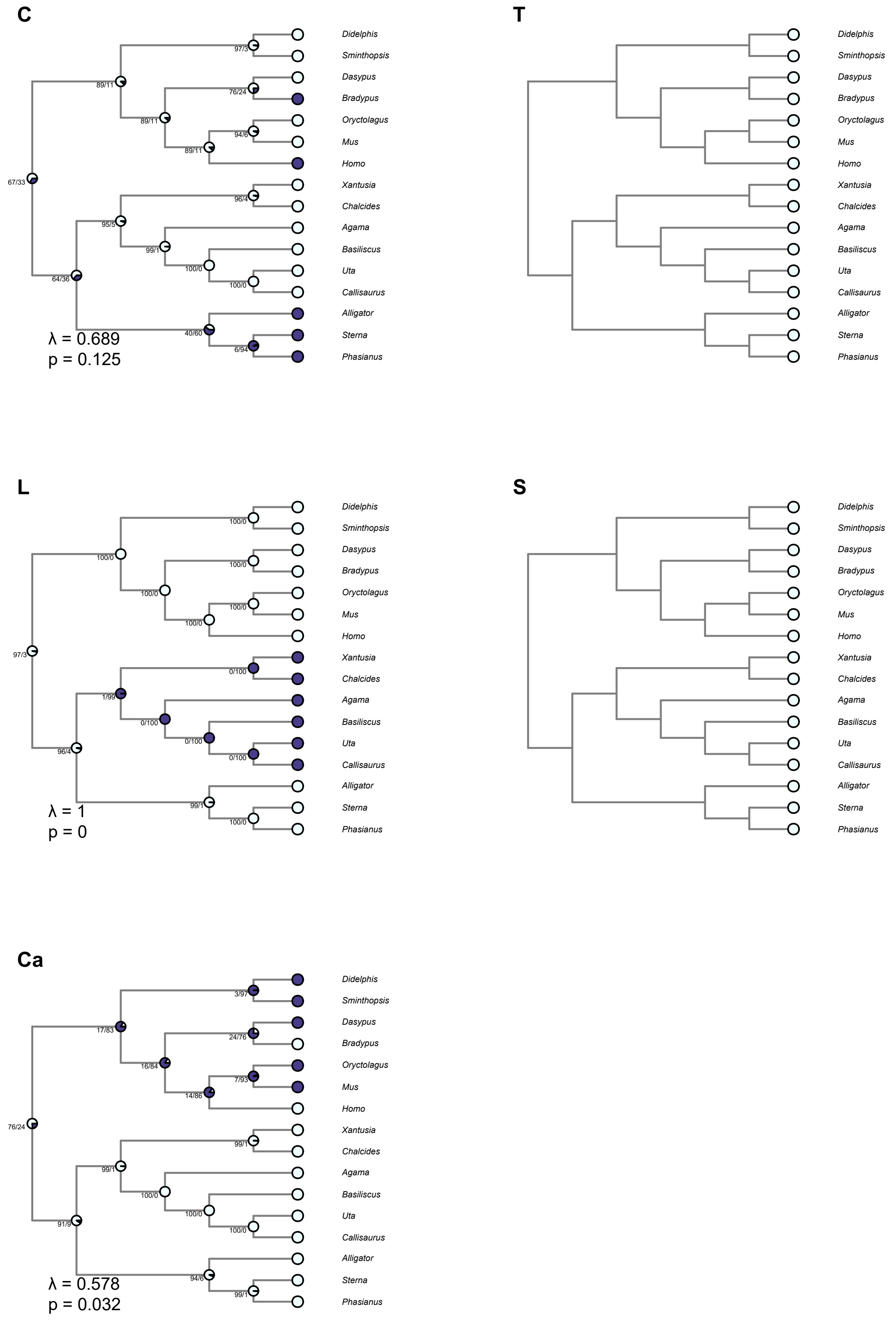

NCF - Mesosaurus: removed - maximum likelihood

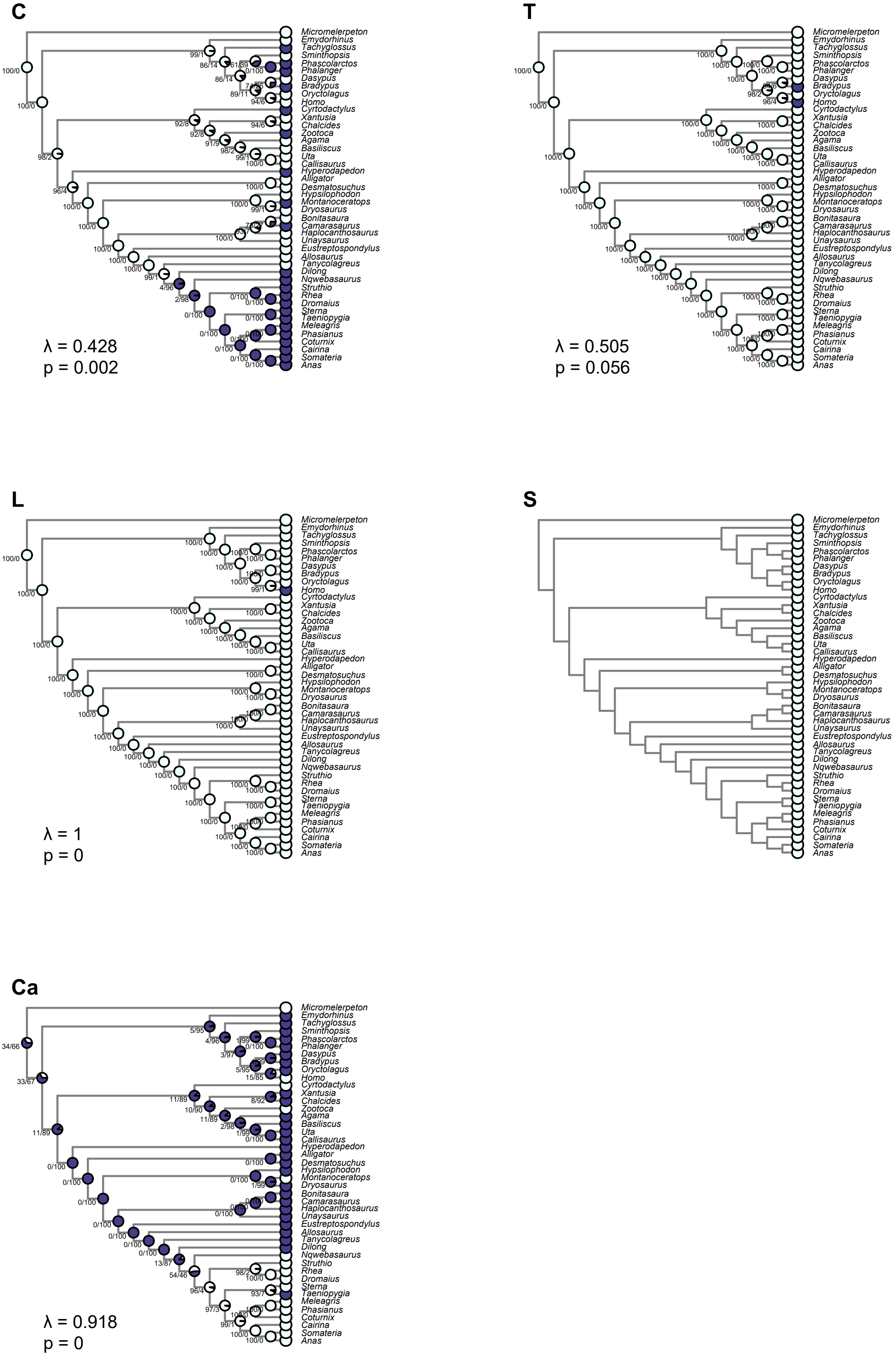

Supplement: Supplementary file 6 — Supplementary Figure S5. [file 41598_2022_24983_MOESM6_ESM.pdf]
